# THE ESOPHAGEAL, GASTRIC, AND COLORECTAL TUMORS AND THE ESOPHAGOGASTRODUODENOSCOPIES AND COLONOSCOPIES BY THE BRAZILIAN UNIFIED HEALTH SYSTEM: WHAT IS THE IMPORTANCE?

**DOI:** 10.1590/0102-672020210002e1661

**Published:** 2022-06-24

**Authors:** Marilisa Ferruda Andreoli RISSO, Luigi Carlo da Silva COSTA, Valdir TERCIOTI, José Antonio Possatto FERRER, Luiz Roberto LOPES, Nelson Adami ANDREOLLO

**Affiliations:** 1 Center for the Diagnosis of the Digestive System Diseases - Gastrocentro - Unicamp, Campinas, São Paulo, Brazil;; 2 Digestive Diseases Surgical Unit, Department of Surgery, Faculty of Medical Sciences, State University of Campinas - Unicamp, Campinas, Sao Paulo, Brazil

**Keywords:** Esophageal neoplasms, Stomach neoplasms, Colorectal neoplasms, Endoscopy, Colonoscopy, Population, Neoplasias esofágicas, Neoplasias gástricas, Neoplasias colorretais, Endoscopias, Colonoscopia, População

## Abstract

Esophagogastroduodenoscopies and colonoscopies are the main diagnostic examinations for esophageal, stomach, and colorectal tumors.

**AIM:**

This study aimed to evaluate the estimates of the incidence of esophageal, stomach, and colorectal cancer; population growth; and esophagogastroduodenoscopies and colonoscopies performed by the Unified Health System (SUS), from 2010 to 2018, in the five regions of the country, and to analyze the relationship between these values.

**RESULTS:**

The colorectal tumor had a significant elevation, while the esophageal and gastric maintained the incidences. In the five regions, there was a significant increase in the number of colonoscopies; however, this increase did not follow the increase in the population in the North and Northeast regions. There was no significant increase in the number of esophagogastroduodenoscopies in the North, Northeast, Midwest, and South regions, and in the North region there was a decrease. In the Northeast region, there was a decreasing number, and in the South and Midwest regions, the number of examinations remained stable in the period. The Southeast region recorded an increase in the number of examinations following the population growth.

**CONCLUSION:**

The current number of esophagogastroduodenoscopies and colonoscopies performed by the SUS did not follow the population growth, in order to attend the population and diagnose esophageal, stomach, and colorectal tumors. Therefore, the country needs to have adequate and strategic planning on how it will meet the demand for these tests and serve the population well, incorporating new technologies.

## RESUMO - RACIONAL

Os tumores de esôfago, estômago e colorretal têm como principal exame diagnóstico as esofagogastroduodenoscopias e colonoscopias.

**OBJETIVO:**

Avaliar as estimativas de incidências de câncer de esôfago, estômago e colorretal, o crescimento populacional, e as esofagogastroduodenoscopias e colonoscopias realizadas pelo Sistema Único de Saúde (SUS) de 2010 a 2018, nas cinco regiões do país e a relação entre esses valores.

**RESULTADOS:**

O tumor colorretal teve elevação significativa, enquanto os esofágicos e gástricos mantiveram as incidências. Nas cinco regiões foi registrado elevação significativa do número de colonoscopias, entretanto, essa elevação não acompanhou a elevação da população nas regiões Norte e Nordeste. Não ocorreu elevação significativa do número de esofagogastroduodenoscopias nas regiões Norte, Nordeste, Centro-Oeste e Sul e na região Norte ocorreu diminuição. Na região Nordeste ocorreu número decrescente e nas regiões Sul e Centro-Oeste o número de exames manteve-se estável no período. A região Sudeste registrou elevação do número de exames acompanhando o crescimento população.

**CONCLUSÃO:**

O número atual de esofagogastroduodenoscopias e colonoscopias realizadas pelo SUS, não acompanhou o crescimento populacional, para atender a população e diagnosticar os tumores de esôfago, estômago e colorretais. Portanto, o país necessita ter um planejamento adequado e estratégico de como irá suprir a demanda desses exames e bem atender a população, incorporando as novas tecnologias.

## INTRODUCTION

In Brazil, as in North America, Europe, and some countries in Asia, esophagus, stomach, and colorectal malignant tumors are among the most frequent cancer in the population, in both sexes, becoming a public health problem, since they involve health services, hospital, and outpatient clinics, of medium and high complexity ^1^ ^,^ ^4^ ^,^ ^12^ ^,^ ^29^.

Esophagogastroduodenoscopies (EGDs) and colonoscopies (CLNs) are the most important examinations for the investigation of disorders of the upper and lower digestive tract and are considered essential for the diagnosis of both benign and malignant lesions. They are also the first option for the diagnosis of gastrointestinal diseases ^5^ ^,^ ^11^ ^,^ ^22^ ^,^ ^30^.

Both EGD and CLN examinations are performed widely by the Unified Health System (SUS), both in an outpatient clinic and in public and university hospitals. EGD offers an excellent view of the mucous surfaces of the esophagus, stomach, and proximal duodenum, and CLN allows the examination of the entire colon and rectum and often the terminal ileum. Both examinations allow diagnostic and therapeutic procedures, including polypectomy, mucous and submucosal resections, dilation of stenosis, placement of stents, removal of foreign bodies, gastrostomy, gastrointestinal bleeding treatments with injection of hemostatic solutions, placement of metal clips, electrocoagulation, laser, sclerotherapy, and elastic bandages of esophageal-gastric varices, in addition to other endoscopic therapies. They are mainly performed on an outpatient clinic, since they are safe procedures, with low morbidity and less complication ^5^ ^,^ ^7^ ^,^ ^13^ ^,^ ^28^ ^,^ ^30^ ^,^ ^31^.

The Brazilian Institute of Geography and Statistics (IBGE) is a government agency responsible for the population control and statistical data. It is also responsible for the organization and execution of the demographic census, which consists of a survey of the national population, by states, and by regions, accumulating data on the number of inhabitants; people’s lives condition; numbers of men, women, adults, and children; and others social and economic information of the country. The last Brazilian census divulged was in 2018 ^21^.

The National Cancer Institute (INCA) is the Ministry of Health’s organ responsible for the development and coordination of integrated actions for the prevention and control of cancer in Brazil. Not only it provides medical and hospital assistance activities to cancer patients, as part of the services offered by SUS, but it also operates in strategic areas, such as prevention and early detection, specialized professionals training, and development of research and epidemiological information. Therefore, it is the main responsibility for cancer statistics in the country to provide detailed information on incidence, occurrence, and mortality of different tumors ^15^.

The objective of this study was to conduct a survey on the websites of the INCA, IBGE, and Government Health Department, to obtain, respectively, the estimates of the incidence of esophageal, stomach, and colorectal cancer; the population growth; and the number of EGDs and CLNs performed by the SUS, from 2010 to 2018, in the five regions of the country (i.e., North, Northeast, Southeast, Midwest, and South), and to analyze the relationship between these values.

## METHODS

The INCA published every 2 years, respectively, in the years 2010, 2012, 2014, 2016, and 2018, reports of the estimated incidence of cancer in the country, in all organs of the human body, as well as by regions ^16^ ^,^ ^17^ ^,^ ^18^ ^,^ ^19^ ^,^ ^20^. Thus, estimates of the incidence of esophageal, stomach, and colorectal cancer were obtained in these years, with the numbers listed by regions and recorded in the figures.

The number of the Brazilian population, by regions, were obtained from the IBGE website ^21^, in the years 2010-2017 (divulged in the 2018 population census), and recorded in the figures.

The number of EGDs and CLNs performed by the SUS, recorded in the site of the Government Health Department, in each of the Federation States, from 2010 and 2017 (divulged in 2018), were obtained ^3^. Based on the number of endoscopic examinations by geographic regions and the population of these regions, the number of examinations per 100,000 inhabitants per year and also by region was calculated, and the result is shown in the table. These numbers were listed by region and recorded in Figures.

Statistical analysis was performed using the computer program SAS System for Windows (Statistical Analysis System), version 9.4 (SAS Institute Inc., 2002-2012, Cary, NC, USA). The Jonckheere-Terpstra (JT) test was used to verify the tendency of an increase in the resident population and outpatient production over the years. To analyze the relationship between population growth and outpatient production in each region, the analysis of the cross-correlation function (CCF) was carried out ^2^ ^,^ ^9^. The level of significance was 5% (p<0.05).

## RESULTS

**Figure 1 -:**
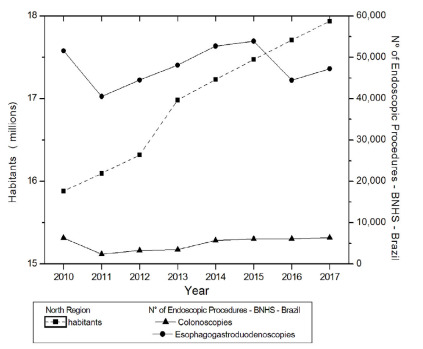
Evolution of the population living in the North and of endoscopic examinations in the same region.

**Figure 2 -:**
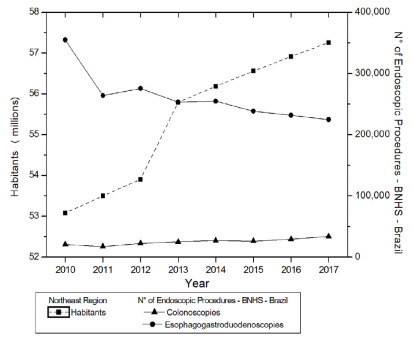
Evolution of the population living in the Northeast region and of endoscopic examinations in the same region.

**Figure 3 -:**
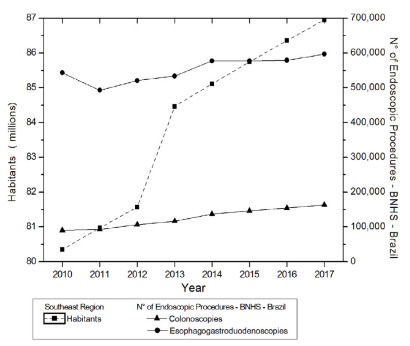
Evolution of the population living in the Southeast region and of endoscopic examinations in the same region.

**Figure 4 -:**
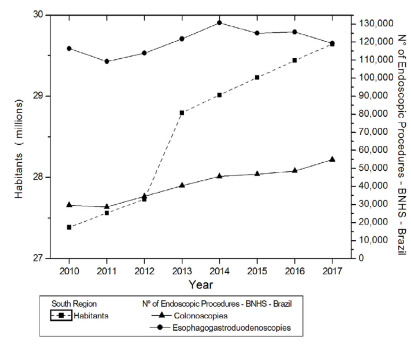
Evolution of the population living in the South and of endoscopic examinations in the same region.

**Figure 5 -:**
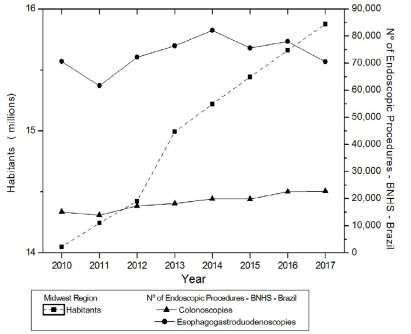
Evolution of the population living in the Midwest region and of endoscopic examinations in the same region.

**Figure 6 -:**
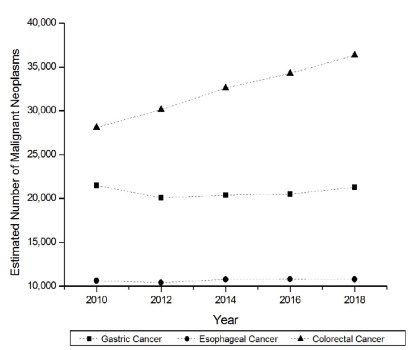
Evolution of the number of esophageal, stomach, and colorectal cancer in the country, from 2010 to 2018.

Figures 1-6 and Table 1 show the findings of this study and analysis.

**Table 1 -:** Number of endoscopic examinations in 2018 per 100,000 habitants, in each region (SUS Outpatient Production - Brazil)

Regions	Number of colonoscopies per 100,00 habitants	Number of esophagogastroduodenoscopies per 100,000 habitants
North	35,453	263,317
Northeast	58,873	392,480
Southeast	187,902	686,427
South	185,155	402,480
Midwest	143,431	444,453

The analysis in Figure 1 shows the evolution of the population living in the North region (millions of inhabitants) and of endoscopic examinations. There was a linear tendency of significant increase for the population (p=0.0005 JT, p<0.05), year by year, from 2010 to 2017. There was a significant upward tendency for the number of CLNs (p=0.0478 JT, p<0.05) and without a tendency to increase the number of EGDs (p=0.6207 JT, p>0.05). The relationship between population growth and endoscopic examinations, carried out by analyzing the CCF, did not show a statistically significant relationship for CLNs (CCF=0.54946; p=0.1202, p>0.05) and for EGDs (CCF=0.22852; p=0.5181, p>0.05).

The analysis in Figure 2 shows the evolution of the population in the Northeast region and of endoscopic examinations, in the same region. There was a linear trend of significant increase for the population (p=0.0005 JT, p<0.05). There was a significant upward trend for CLNs (p=0.0478 JT, p<0.05) and no tendency for EGDs (p=0.6207 JT, p>0.05). The relationship between the population and endoscopic examinations did not show a statistically significant relationship for CLNs (CCF=0.54946; p=0.1202, p>0.05) or for EGDs (CCF=0.22852; p=0.5181, p>0.05).

The analysis in Figure 3 shows the evolution of the population in the Southeast region and of endoscopic examinations, in the same region. There was a linear trend of significant statistical increase for the population (p=0.0005 JT, p<0.05). The trend of significant increase for CLNs (p=0.0005 JT, p<0.05) and for EGDs (p=0.0133 JT, p<0.05) was recorded. The relationship between the population and endoscopic examinations showed a statistically significant relationship for CLNs (CCF=0.97196; p=0.0060, p<0.05) and for EGDs (CCF=0.84408; p=0.0170, p<0.05).

The analysis in Figure 4 shows the evolution of the population in the South and of endoscopic examinations, in the same region. There was a linear trend of significant statistical increase for the population (p=0.0005 JT, p<0.05). There was a significant upward trend for CLNs (p=0.0013 JT) and no upward trend for EGDs (p=0.1376 JT, p>0.05). The relationship between the series of population and examinations showed a statistically significant relationship for CLNs (CCF=0.97778; p=0.0057, p<0.05) and for EGDs (CCF=0.74148; p=0.0360, p<0.05).

The analysis in Figure 5 shows the evolution of the population in the Midwest region and of endoscopic examinations, in the same region. There was a linear trend of significant statistical increase for the population (p=0.0005 JT, p<0.05). There was a trend of significant statistical increase for CLNs (p=0.0030 JT, p<0.05) and without an increase trend for EGDs (p=0.3223 JT, p>0.05). The relationship between population and examinations showed a statistically significant relationship only for CLNs (CCF=0.96125; p=0.0066, p<0.05) and not for EGDs (CCF=0.53031; p=0.1336, p>0.05).

The analysis in Figure 6 shows the general frequency of esophageal, stomach, and colorectal malignancies in the country, between 2010 and 2018. There was no linear trend of significant statistical increase for gastric cancer (p=0.6242 JT, p>0.05) and esophageal cancer (p=0.1416 JT, p>0.05). However, in the same period, there was a linear trend of increase with statistical significance for colorectal cancer (p=0.0143 JT, p<0.05).

Therefore, these results show that in all regions of the country, there was a statistically significant increase in population, between the years 2010 and 2018. The colorectal tumor had a statistically significant increase in the country; however, in the same period, esophageal and gastric tumors did not increase. In the five regions of the country, there was a significant increase in the number of colonoscopies performed from 2010 to 2017; however, this increase did not follow the increase in the population in the North and Northeast regions.

The statistical analyses performed showed that there was no significant increase in the number of EGDs in the North, Northeast, Midwest, and South region. In the North region, there was a decrease in the number of these examinations in the same period. In the Northeast region, a decreasing number has been recorded since 2010. In the South and Midwest regions, the number of examinations remained constant. Therefore, in these regions, the number of examinations performed has not followed the same population growth, which has been increasing year by year. On the contrary, in the Southeast region, there was an increase in the number of EGDs, followed by the population growth, with a statistically significant relationship.

The number of endoscopic examinations per 100,000 habitants, in each region, in 2018, varied in the country, as shown in Table 1.

Therefore, the number of EGDs per 100,000 habitants varied from region to region, in decreasing order, respectively, in the Southeast, Midwest, South, Northeast, and North regions. The number of CLNs per 100,000 habitants per year also varied from region to region, in decreasing order, respectively, in the Southeast, South, Midwest, Northeast, and North regions.

## DISCUSSION

The incidence and statistics of malignant tumors of the esophagus, stomach, and colorectal in the Brazilian population are high and significant ^1^ ^,^ ^15^ ^,^ ^29^. The most frequent malignant esophageal tumors are squamous cell carcinomas, followed by adenocarcinomas, which affect the esophagogastric junction. In Brazil, 8,690 cases in men and 2,700 in women are awaited to be diagnosed for each year of the 2020-2022 triennium, corresponding to an estimated risk of 8.32 new cases for every 100,000 men and 2.49 for every 100,000 women. Among men, in the Northeast (5.58/100,000 habitants) and Midwest (6.64/100,000 habitants) regions, these tumors occupy the sixth position, among tumors in general. In the North region (2.69/100,000 habitants), it is the eighth most frequent neoplastic disease, and in the Southeast region (9.53/100,000 habitants), it occupies the seventh position. In the South region (14.48/100,000 habitants), they occupy the fifth position, among the other tumors. Among women, in the North region (0.73/100,000 habitants), they occupy the 14th position, in the Northeast region (2.30/100,000 habitants) and the South region (4.52/100,000 habitants) occupying the 13th position, and in the Southeast (2.39/100,000 habitants) and Midwest (1.96/100,000 habitants) regions occupying the 15th position among the other tumors. Mortality in Brazil in 2017 was 6,647 cases, with a gross mortality rate of 6.58 of 100,000 men and 1,907 cases, with a crude rate of 1.84 of 100,000 women ^15^.

The most frequent malignant gastric tumors are adenocarcinomas (95%), and in Brazil, 13,360 new cases are awaited in men and 7,870 in women for each year of the 2020-2022 triennium, with an estimated risk of 12.81 for every 100,000 men and 7.34 for every 100,000 women. Among men, in the North (11.75/100,000 habitants), it is the second most frequent, followed by the Northeast (10.63/100,000 habitants) occupying the third position, while in the South (16.02/100,000 habitants), Southeast (13.99/100,000 habitants), and Midwest (9.38/100.000 habitants), it is the fourth most frequent among the other tumors. Among women, in the North (6.03/100,000 habitants) and South (9.15/100,000 habitants) regions, it is the fifth most frequent; in the Northeast (7.03/100,000 habitants) and Central-West regions (6.71/100,000 habitants), it occupies the sixth position; and in the Southeast region (7.30/100,000 habitants), it occupies the seventh position. In Brazil, in 2017, there were 9,206 deaths from gastric cancer in men and 5,107 deaths in women, corresponding to the risk of 9.12 and 4.93 per 100,000 habitants ^15^.

The most frequent colorectal malignancies (divided into colon and rectum) are adenocarcinomas, and in Brazil, it is estimated that for each year of the 2020-2022 triennium, 20,520 cases of colorectal cancer in men and 20,470 in women will be diagnosed, corresponding to an estimated risk of 19.63 new cases per 100,000 men and 19.03 for every 100,000 women. Among men, in the North (5.27/100,000 habitants) and Northeast (8.91/100,000 habitants) regions, they occupy the fourth position; in the South region (25.11/100,000 habitants), it is the third most frequent tumor; and in the Southeast (28.62/100,000 habitants) and Midwest (15.40/100,000 habitants) regions, it is the second most incident. For women, in the Northeast (10.79/100,000 habitants), North (6.48/100,000 habitants), and Midwest (15.24/100,000 habitants) regions, it is the third most incident; and in the Southeast (26.18 /100,000 habitants) and South (23.65/100,000 habitants) regions, it is the second most frequent. In Brazil, in 2017, there were 9,207 deaths from colon and rectal cancer, respectively, in men and women, to the risk of 9.12 and 9.33 per 100,000 habitants ^15^.

Both EGDs and CLNs have many indications among gastroenterologists, digestive surgeons, general surgeons, proctologists, and general practitioners. They are safe procedures with low morbidity and are used for the diagnosis of benign and malignant diseases and for therapeutics ^13^ ^,^ ^14^ ^,^ ^23^ ^,^ ^24^.

The Unified Health System (SUS) records the number of examinations performed in the country, state by state. Consulting the online available records, it was possible to construct the figures shown in this study, comparing and statistically analyzing the number of endoscopic examinations performed, with the population growth recorded by IBGE in the geographic regions and the regional incidence of these malignant tumors, recorded by INCA, year by year.

The Brazilian Constitution considers health as a universal right and a governmental responsibility to every citizen, through an SUS, created in 1990, as a health service, throughout the country, for everyone. Thus, SUS allows the population to access the outpatient and hospital health system, from primary care to highly complex treatments ^6^.

The population senses, performed by the IBGE, recorded population growth in the five Brazilian regions between 2010 and 2017 (Figures 1-5). In contrast, INCA recorded, between 2010 and 2018, a significant increase in colorectal tumors and incidence of stable esophageal and gastric tumors (Figure 6).

The results showed that the number of EGDs performed in the North, Northeast, South, and Midwest regions did not increase significantly during the period of this study. In the Northeast region, the number of examinations decreased year by year. Therefore, the number of examinations did not follow the population growth. Only in the South region, did the number of examinations increase, following the population growth (Table 1).

The number of CLNs increased year by year in the five regions of the country. However, in the North and Northeast regions, population growth was more significant and the number of CLN examinations performed did not follow the population growth (Figures 1 and 2).

Although malignant gastric tumors have decreased in some countries, in recent years, such as Japan, Korea, and China, their incidence remains high, requiring health actions for screening in the population, based on encouraging EGDs. In Japan, there was an increase in the number of examinations per year, by more than 2 million from 1996 to 2014. And from 2016, the government started to encourage the requesting of an increasing number of EGDs in clinics and hospitals, achieving an increase of 8.6% per year, and that a total of 12 million examinations be performed annually, for an estimated population in 2019, of 126 million habitants ^14^.

In China, with 1.4 billion habitants in 2019, it was estimated that approximately 2 million Chinese people had malignant gastric tumors, and of this total, 42.7% (40.3-45.0%) remained without diagnosis, due to patients’ delay in seeking medical help and difficulty in performing EGDs. In addition, without systematic screening, it is projected to occur about 10 million cases and 7 million deaths, due to this tumor, in the next 30 years. The authors concluded that the triennial screening, performing periodic EGDs in the population at risk, would gradually reduce by 38.8% (36.9-40.7%), 25.5% (23.4-27.6%), and 17.8% (16.0-19.6%) by 2049, respectively, the proportion of undiagnosed cases of gastric cancer ^8^.

In Germany, 36.6% of deaths from colorectal cancer are estimated to be attributed to the failure to perform CLNs, this percentage being compared to what also occurs in the United States, where it is estimated that 38.2% and 33.6%, respectively, between the years 2008-2009 and 2010-2011. The authors concluded that the proportion of deaths theoretically that could be prevented in 10 years would be 30.7% and 29%, respectively, in Germany and the United States, in these cited periods ^7^.

The cost-effectiveness for detecting malignant esophagogastric junction and gastric tumors in the United States, in a high-risk population, using EGDs, was considered advantageous, in relation to the cost of surgical and oncological treatment of these tumors when diagnosed in advanced phase ^26^ ^,^ ^29^.

Hamashima emphasized that endoscopic examinations are the main methods for diagnosing gastric tumors, and the sensitivity and specificity of endoscopic screening evaluated in South Korea and Japan. Although the reduction in mortality employing endoscopic screening was recorded in several studies, the results remain insufficient to confirm its effectiveness, and so new screening methods should be introduced in future ^14^.

The literature has recorded high rates of colorectal cancer in several countries, including Brazil, and evidenced colonoscopy is the best and most effective screening test. Therefore, maximizing its effectiveness and encouraging its performance in adults over 50 years of age is the best way to detect the tumor early and provide an opportunity for appropriate treatment for the patient ^5^ ^,^ ^10^ ^,^ ^25^ ^,^ ^27^.

Taveira et al. calculated the number of EGDs per 100,000 inhabitants per year, for the period from January 2008 to December 2009, analyzing the total Brazilian population according to IBGE data and the population, in the respective states and regions. The authors concluded that the regions that performed the most EGDs in absolute number per year, in decreasing order, were Southeast, Northeast, South, Midwest, and North. On the contrary, in these 2 years of study, calculating the number of examinations per 100 thousand inhabitants per year, the regions that performed more examinations, in decreasing order, were Northeast (654 examinations), Southeast (590 examinations), Midwest (451 examinations), South (409 examinations), and North (323 examinations), with the median being 550 examinations per 100,000 habitants per year. By comparing this national average with countries like Ireland, Holland, and England that have health systems similar to SUS and performed 1,322, 1,137, and 950 examinations per 100,000 habitants per year, respectively, they found that Brazil performed half of the examinations than these countries. The authors concluded that adequate planning is necessary to incorporate new diagnostic technologies in order to serve the population of all regions of the country well ^28^.

Comparing the results obtained by Taveira et al. ^28^, in 2009, with those recorded in this study in 2018, it can be concluded that occurred population growth in all geographic regions, however, the number of EGDs did not follow this growth in the same proportion.

Patients looking for medical help present these tumors in an advanced form; so it is important to implement specific health actions to provide orientations to the population, encouraging the early search for Medical Services and requesting these endoscopic examinations, for the diagnosis the tumors in early stages. The diagnosis of these tumors in early and non-advanced stages allows greater chances of surgical and oncological treatments, making possible better disease control and increased survival and quality of life ^1^ ^,^ ^14^ ^,^ ^29^.

Therefore, it is of great importance to know quantitative data of these examinations, to plan health actions, mainly in the malignant tumors of the digestive system, because of what they represent in the Brazilian population.

## CONCLUSION

The current number of EGDs and CLNs performed by the SUS did not follow the population growth, to attend the population and diagnose esophageal, stomach, and colorectal tumors. Therefore, an adequate and strategic planning of how to attend the demand for endoscopic examinations is important, in all regions, incorporating new technologies, in the diagnosis of these tumors, in future.
